# Performance of Body Fat Percentage, Fat Mass Index and Body Mass Index for Detecting Cardiometabolic Outcomes in Brazilian Adults

**DOI:** 10.3390/nu15132974

**Published:** 2023-06-30

**Authors:** Bianca Rodrigues de Oliveira, Elma Izze da Silva Magalhães, Maylla Luanna Barbosa Martins Bragança, Carla Cristine Nascimento da Silva Coelho, Natália Peixoto Lima, Heloisa Bettiol, Marco Antônio Barbieri, Viviane Cunha Cardoso, Alcione Miranda dos Santos, Bernardo Lessa Horta, Antônio Augusto Moura da Silva

**Affiliations:** 1 Postgraduate Programme in Collective Health, Federal University of Maranhão, São Luís 65020-070, MA, Brazil; elmaizzenutri@gmail.com (E.I.d.S.M.); mayllabmartins@gmail.com (M.L.B.M.B.); carlacristinecoelho@gmail.com (C.C.N.d.S.C.); alcione.miranda@gmail.com (A.M.d.S.); aamouradasilva@gmail.com (A.A.M.d.S.); 2 Postgraduate Programme in Epidemiology, Federal University of Pelotas, Pelotas 96020-220, RS, Brazil; natyplima@hotmail.com (N.P.L.); blhorta@gmail.com (B.L.H.); 3 Postgraduate Programme in Child and Adolescent Health, University of São Paulo, Ribeirão Preto 14048-900, SP, Brazil; hbettiol@fmrp.usp.br (H.B.); mabarbieri@fmrp.usp.br (M.A.B.); vicuca@fmrp.usp.br (V.C.C.)

**Keywords:** body fat percentage, fat mass index, body mass index, cut-off, ROC curve, cardiometabolic risk factors

## Abstract

Obesity is a recognized risk factor for the development of cardiometabolic outcomes. Therefore, it is essential to evaluate anthropometric and body composition indicators used for its diagnosis. This study aimed to assess the diagnostic performance of body fat percentage (BF%), fat mass index (FMI) and body mass index (BMI) for detecting cardiometabolic outcomes in adults. A cross-sectional study was conducted involving adults at 30 years of age from Pelotas, RS (n = 3517) and at 37–39 years from Ribeirão Preto, SP (n = 1696). Receiver operating characteristic (ROC) curves were used to determine the cut-off points for predicting cardiometabolic risk factors, including altered blood pressure, blood glucose, triglycerides, total cholesterol, low-density lipoprotein cholesterol (LDL-c), high-density lipoprotein cholesterol (HDDL-c), C-reactive protein and glycated hemoglobin. The cut-off points of BF% ranged from 25.2 to 27.8 in men and from 37.4 to 39.7 in women at 30 years, and from 26.1 to 27.8 in men and from 38.5 to 42.2 in women at 37–39 years. For FMI (kg/m^2^), the cut-off points ranged from 6.3 to 7.5 in men and from 9.5 to 10.8 in women at 30 years, and from 7.3 to 7.8 in men and from 10.2 to 12.2 in women at 37–39 years. The BMI cut-off points (kg/m^2^) ranged from 26.3 to 27.3 in men and from 25.4 to 27.2 in women at 30 years, and from 28.3 to 29.0 in men and from 27.2 to 29.6 in women at 37–39 years. The areas under the curve were similar for the three indicators, ranging from 0.523 to 0.746. BMI showed a performance similar to that of the body fat-based indicators in identifying cardiometabolic outcomes. The cut-off points of the three indicators showed acceptable discriminatory power in subjects with cardiometabolic risk factors.

## 1. Introduction

Obesity is a progressive chronic disease with multifactorial and complex etiology [1]. It is defined as abnormal or excessive body fat accumulation that may impair health [2]. The global prevalence of obesity has increased from 7% in 1980 to 12.5% in 2015, an increase of almost 80%, reaching pandemic levels [1]. In Brazil, the prevalence of obesity in adults increased from 11.8% in 2006 to 20.3% in 2019, corresponding to an annual increase of 3.8% [3].

Obesity contributes to incidence of cardiovascular risk factors, including dyslipidemia, type 2 diabetes and hypertension [4] via multiple direct and indirect pathophysiological mechanisms [5]. In summary, increased overall body adiposity causes multiple cardiovascular pathological disorders, concerning electrocardiographic, hemodynamic, structural and functional changes. Such chronic alterations increase the risk of cardiovascular diseases, as well as related adverse cardiometabolic complications [6]. Moreover, obesity increases healthcare costs, reduces work productivity and quality of life and causes disability and premature death [1].

Although it is not a direct measure of body fat, the body mass index (BMI) is the parameter most frequently used for the diagnosis of obesity in clinical practice and in population studies [7]. A BMI equal to or greater than 30 kg/m^2^ is widely used to classify obesity [2]. Despite criticism for being unable to distinguish between fat and lean mass, Ortega et al. [8] found that BMI was a strong predictor of cardiovascular disease compared to accurate measurements of body fat.

In fact, BMI as a proxy measure of body fat can erroneously classify an individual with a high body fat percentage (BF%) but with normal weight (18.5 to 24.9 kg/m^2^) as “non-obese”, potentially missing the opportunity to prevent or treat excess body fat and associated cardiometabolic risk factors [9]. Within this context, BF% may be a more suitable indicator for detecting obesity [10], as it has been associated with metabolic deregulation regardless of body weight [11]. However, there is no universally accepted definition of obesity based solely on body fat content [12].

Another indicator for diagnosing obesity is the fat mass index (FMI) proposed by VanItallie et al. [11]. It is obtained by dividing fat mass in kilograms by the square height in meters. This indicator eliminates BF% differences associated with height and may be useful for identifying obesity [13]. Individuals with the same height can have different BF% values since the latter depends on the content of lean mass. As BF% includes fat mass in both the numerator and the denominator, its interpretation as a measure of body fat is limited. The ideal approach would be to adjust for a measure of body size not related to fat mass, such as height [14,15]. Additionally, the FMI has been suggested to be a better indicator than BMI or BF% when screening for metabolic syndrome [13].

Given the importance of early detection of obesity for implementing intervention measures [7], it is essential to evaluate the indicators used for the diagnosis of obesity. Therefore, the aim of the present study was to assess the diagnostic performance of BF%, FMI and BMI in detecting cardiometabolic risk factors in adults from two Brazilian cities.

## 2. Materials and Methods

### 2.1. Study Design and Participants

This cross-sectional study used data from two Brazilian birth cohorts initiated in the cities of Ribeirão Preto, São Paulo, in 1978/79 and in Pelotas, Rio Grande do Sul, in 1982. Participants from both cohorts have been followed up with at various time points since birth. For this study, data from the 1982 Pelotas cohort obtained at 30 years of age and of the 1978/79 Ribeirão Preto cohort obtained at 37–39 years were utilized. Methodological details of these cohorts have been described elsewhere [16,17].

**Figure 1:**
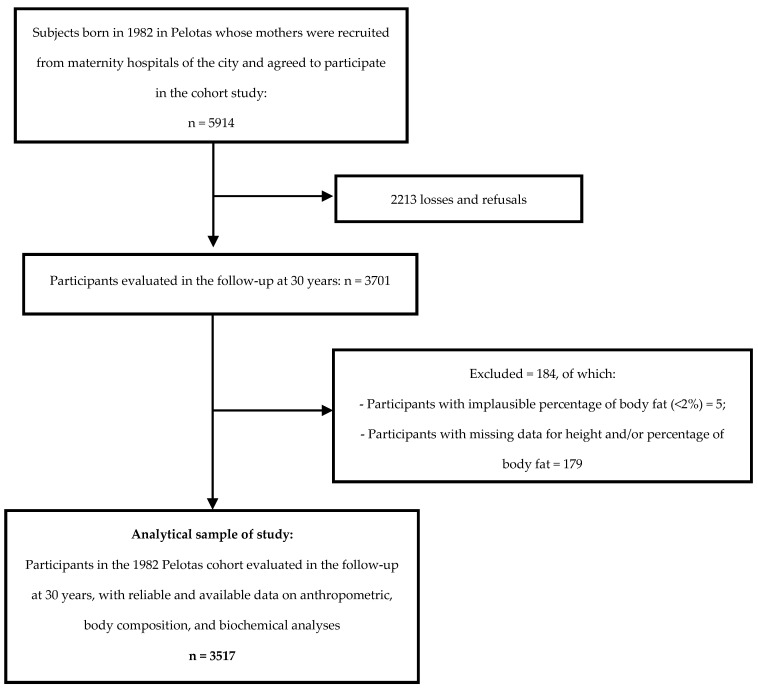
Flowchart of participants from the 1982 Pelotas cohort evaluated in the follow-up at 30 years included in this study. Pelotas (RS), 2012/2013.

In the 1982 Pelotas cohort, the 30-year follow-up was conducted between June 2012 and February 2013, with an attempt to include all 5914 subjects from the initial cohort. A total of 3701 participants were interviewed, and, along with the 325 known deaths, this represented a follow-up rate of 68.1% [16,17]. The present study included data from 3517 adults after exclusion of subjects with implausible BF% (less than 2%; n = 5) and those with missing data (n = 179) (Figure 1).

**Figure 2:**
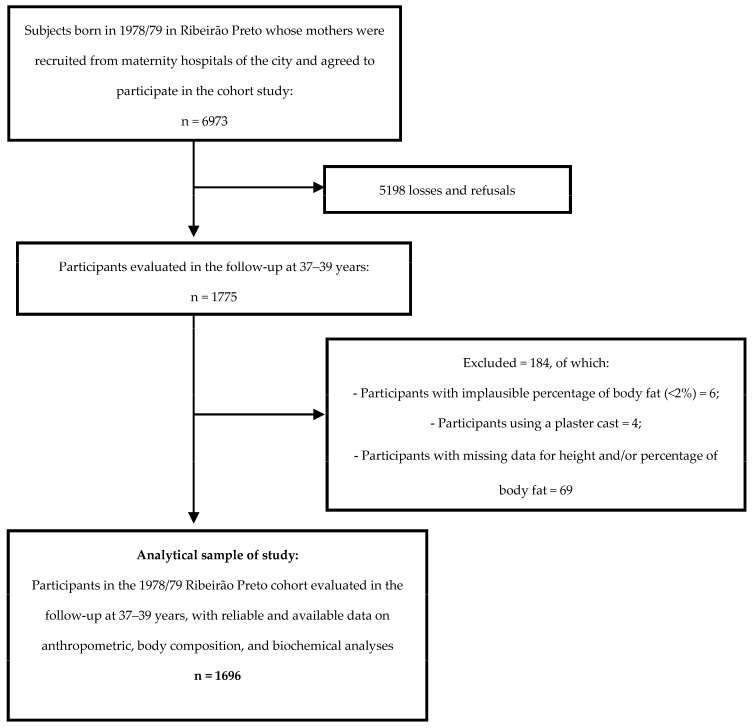
Flowchart of participants from the 1978/79 Ribeirão Preto cohort evaluated in the follow-up at 37–39 years included in this study. Ribeirão Preto (SP), 2016/2017.

In the 1978/79 Ribeirão Preto cohort, an attempt was made in 2016/2017 to include all members of the original cohort (age 37 to 39 years). Out of the 6973 livebirths followed up with during the first phase of the cohort, 1775 were located and evaluated, representing 25.5% of the original cohort [17]. Subjects with BF% less than 2% (n = 6), subjects using a plaster cast (n = 4) and subjects with missing data (n = 69) were excluded from the present study, resulting in a final sample of 1696 adults (Figure 2).

Anthropometric, body composition and biochemical measurements were collected during the follow-up at 30 years in the 1982 Pelotas cohort and the follow-up at 37–39 years in the 1978/79 Ribeirão Preto cohort, as described posteriorly.

### 2.2. Ethical Aspects

The Ribeirão Preto study was approved by the Research Ethics Committee of the University Hospital of the Ribeirão Preto School of Medicine, University of São Paulo (Approval number 1.282.710). The study conducted in Pelotas was approved by the Ethics Committee of the Faculty of Medicine, Federal University of Pelotas (Approval number 16/12). All participants signed the informed consent form.

### 2.3. Anthropometric and Body Composition Measures

Trained professionals collected the data at both centers. In Pelotas, body weight (kg) was measured with a scale coupled to a Bod Pod^®^, while in Ribeirão Preto, a Filizola^®^ scale was used. Height (cm) was measured with an Alturexata^®^ stadiometer in both cohorts. The anthropometric measurements followed techniques recommended by the WHO [18]. The BMI (kg/m^2^) was calculated as weight (kg) divided by the square of the height (m^2^). Obesity was defined as a BMI ≥ 30.0 kg/m^2^ [2]. In both cities, an air displacement plethysmography system (Bod Pod^®^ Gold Standard, COSMED USA, Inc., Concord, CA, USA) was used to measure fat mass and BF%. BF% was estimated using the Siri equation (1961) [19]. The FMI (kg/m^2^) was obtained by dividing fat mass (kg) by the square of the height (m^2^) [11].

### 2.4. Cardiometabolic Risk Factors

In Pelotas, the blood pressure was measured on the left arm with a digital sphygmomanometer (Omron HEM 705 CPINT^®^) using a cuff which was specific for obese individuals. Blood pressure was measured twice, and the mean of the two measurements was used in the analyses. Blood glucose, total cholesterol, high-density lipoprotein cholesterol (HDL-c), low-density lipoprotein cholesterol (LDL-c) and triglycerides were measured by an enzymatic calorimetric method in an automated Mindray^®^ BS-380 Chemistry Analyzer (Shenzhen Mindray Bio-Medical Electronics Co., Ltd., Shenzhen, China). Percentage of glycated hemoglobin was determined by high-performance liquid chromatography (HPLC) combined with ion-exchange chromatography in a Bio-Rad^®^ system. C-reactive protein was analyzed by turbidimetry in an automated analyzer (Mindray^®^ BS-380).

In Ribeirão Preto, blood pressure was measured on both arms with a semi-automatic Omron HEM 742INT^®^ blood pressure monitor and the measurement of the arm providing the higher value was recorded. This procedure was conducted in triplicate and the mean of the three measurements was considered. Total cholesterol, HDL-c, triglycerides and blood glucose were measured by automated biochemistry (Weiner, Rosario, Argentina). LDL-c was obtained using the equation of Friedwald. Glycated hemoglobin was measured using the same method and equipment as in Pelotas. Concentration of C-reactive protein was measured by a calorimetric method with a Wiener SMD 820I^®^ system in 2016 and with a Wiener CT 600I^®^ system in 2017.

The laboratory tests in the two cities were not performed after fasting since the extensive collection of different data did not allow fasting of the participants for the biochemical analyses.

The cardiometabolic risk factors were classified as altered considering the following cut-off values: systolic blood pressure ≥ 130 mmHg and/or diastolic blood pressure ≥ 85 mmHg or use of antihypertensive medication; blood glucose ≥ 100 mg/dL or use of antihyperglycemic medication [20]; triglycerides ≥ 175 mg/dL or use of lipid-lowering drugs; total cholesterol ≥ 190 mg/dL or use of lipid-lowering drugs; LDL-c ≥ 160 mg/dL or use of lipid-lowering drugs; HDL-c < 40 mg/dL for men and < 50 mg/dL for women or use of lipid-lowering drugs; C-reactive protein > 2 mg/dL [21] and glycated hemoglobin ≥ 5.7% or use of antihyperglycemic medication [22].

### 2.5. Data Analysis

The diagnostic performance of BF%, FMI and BMI was assessed by calculating sensitivity, specificity and constructing receiver operating characteristic (ROC) curves. ROC curves were used to identify cut-off points for BF%, FMI and BMI that provided the best balance between sensitivity and specificity in detecting cardiometabolic risk factors (blood pressure, blood glucose, triglycerides, total cholesterol, LDL-c, HDL-c, C-reactive protein, glycated hemoglobin and cluster ≥ 3 risk factors). The area under the ROC curve (AUC) was calculated, with an AUC of 1 indicating a perfect diagnostic test. An AUC of 0.5 indicates no discrimination, 0.7 ≤ AUC < 0.8 acceptable discrimination, 0.8 ≤ AUC < 0.9 excellent discrimination and > 0.9 outstanding discrimination [23]. Differences between the AUC of BF%, FMI and BMI for each cardiometabolic risk factor were evaluated by comparing the curves.

The data were analyzed with the Stata^®^ 14.0 software (Stata Corp, College Station, TX, USA). All analyses were stratified by cohort and sex. Box plots, histograms and skewness and kurtosis coefficients were used to evaluate the distribution of continuous variables. Continuous variables showing a normal distribution were reported as the mean and 95% confidence interval (95% CI), and those showing a non-normal distribution as median and interquartile range. Differences in the continuous variables between sexes were tested using the Student’s *t*-test for normally distributed variables or the nonparametric Mann–Whitney test for non-normally distributed variables. Categorical variables are described as absolute and relative frequencies and differences between sexes were evaluated using Pearson’s chi-squared test.

## 3. Results

### 3.1. Subjects

Among the 30-year-old adults from Pelotas, 1735 men and 1782 women were evaluated. The median BMI was 26.3 kg/m^2^ in men and 25.3 kg/m^2^ in women (*p* < 0.001), while the FMI was 6.5 kg/m^2^ in men and 9.5 kg/m^2^ in women (*p* < 0.001). Men had a mean BF% of 24.6% whereas women had 37.4% (*p* < 0.001). The prevalence of altered blood pressure, blood glucose, triglyceride (*p* < 0.001), total cholesterol (*p* = 0.007) and LDL-c (*p* = 0.005) values was higher in men compared to women. The prevalence of three or more cardiometabolic risk factors was 26.1% in men and 12.1% in women (*p* < 0.001) (Table 1).

Among the adults from Ribeirão Preto aged 37–39 years, there were 808 men and 888 women. In men, the median BMI was 28.3 kg/m^2^, whereas in women it was 27.4 kg/m^2^ (*p* = 0.001). The median FMI was 7.5 kg/m^2^ in men and 10.5 kg/m^2^ in women (*p* < 0.001), while the mean BF% was 25.9% in men and 38.3% in women (*p* < 0.001). Men had a higher prevalence of altered blood pressure, blood glucose, triglycerides, total cholesterol (*p* < 0.001), LDL-c (*p* = 0.003) and glycated hemoglobin (*p* = 0.025). On the other hand, women had a higher prevalence of reduced HDL-c (*p* < 0.001) and altered C-reactive protein (*p* = 0.001). The prevalence of having three or more cardiometabolic risk factors was 47.8% in men and 27.4% in women (*p* < 0.001) (Table 1).

### 3.2. Diagnostic Performance of BF%, FMI and BMI to Detect Cardiometabolic Risk Factors

In adults from Pelotas, the cut-off points for identifying cardiometabolic risk factors in men ranged from 25.2% (AUC: 0.655) to 27.8% (AUC: 0.666) for BF%, from 6.3 kg/m^2^ (AUC: 0.523) to 7.5 kg/m^2^ (AUC: 0.660) for FMI and from 26.3 kg/m^2^ (AUC: 0.626) to 27.3 kg/m^2^ (AUC: 0.625) for BMI. In women, these values ranged from 37.4% (AUC: 0.600) to 39.7% (AUC: 0.638) for BF%, from 9.5 kg/m^2^ (AUC: 0.595) to 10.8 kg/m^2^ (AUC: 0.679) for FMI and from 25.4 kg/m^2^ (AUC: 0.583) to 27.2 kg/m^2^ (AUC: 0.673) for BMI (Table 2).

In adults from Ribeirão Preto, the cut-off points in men ranged from 26.1% (AUC: 0.651) to 27.8% (AUC: 0.614) for BF%, from 7.3 kg/m^2^ (AUC: 0.661) to 7.8 kg/m^2^ (AUC: 0.622) for FMI and from 28.3 kg/m^2^ (AUC: 0.672) to 29.0 kg/m^2^ (AUC: 0.624) for BMI. In women, these values ranged from 38.5% (AUC: 0.604) to 42.2% (AUC: 0.740) for BF%, from 10.2 kg/m^2^ (AUC: 0.620) to 12.2 kg/m^2^ (AUC: 0.740) for FMI and from 27.2 kg/m^2^ (AUC: 0.626) to 29.6 kg/m^2^ (AUC: 0.738) for BMI (Table 2).

### 3.3. Comparison of the AUC Values of BF%, FMI and BMI to Detect Cardiometabolic Risk Factors

**Figure 3:**
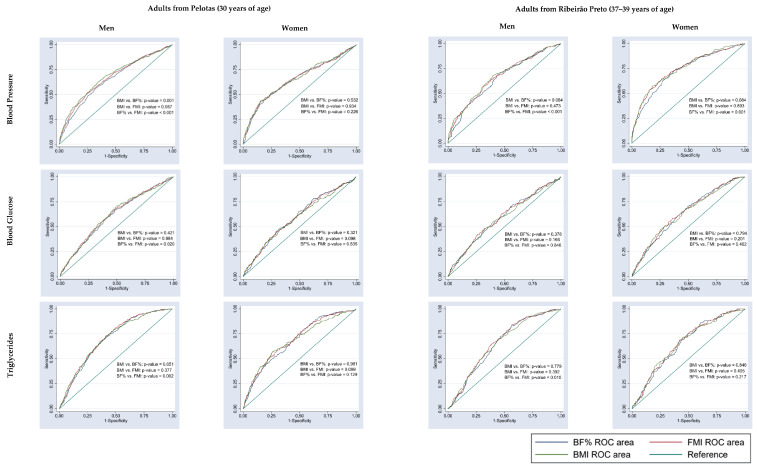
Comparison of the AUC values of body fat percentage (BF%), fat mass index (FMI) and body mass index (BMI) to predict cardiometabolic risk factors (blood pressure, blood glucose and triglycerides) in adults from Pelotas (RS), 2012/2013, and from Ribeirão Preto (SP), 2016/2017.

**Figure 4:**
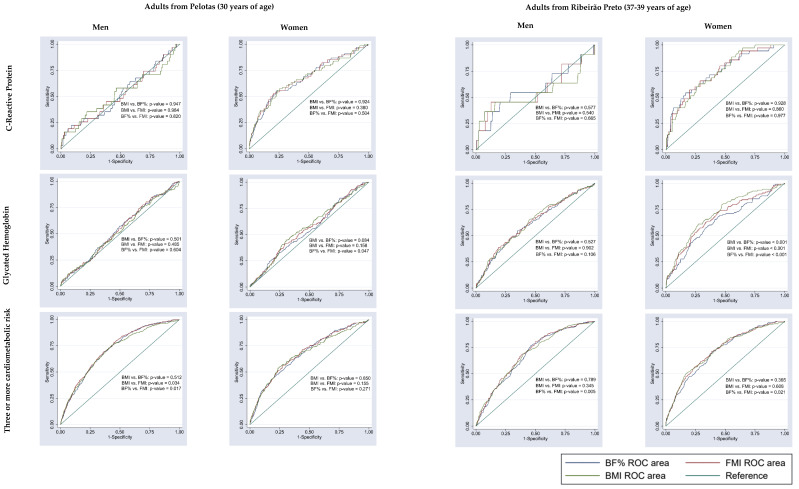
Comparison of the AUC values of body fat percentage (BF%), fat mass index (FMI) and body mass index (BMI) to predict cardiometabolic risk factors (C-reactive protein, glycated hemoglobin and three or more cardiometabolic risk factors) in adults from Pelotas (RS), 2012/2013, and from Ribeirão Preto (SP), 2016/2017.

**Figure 5:**
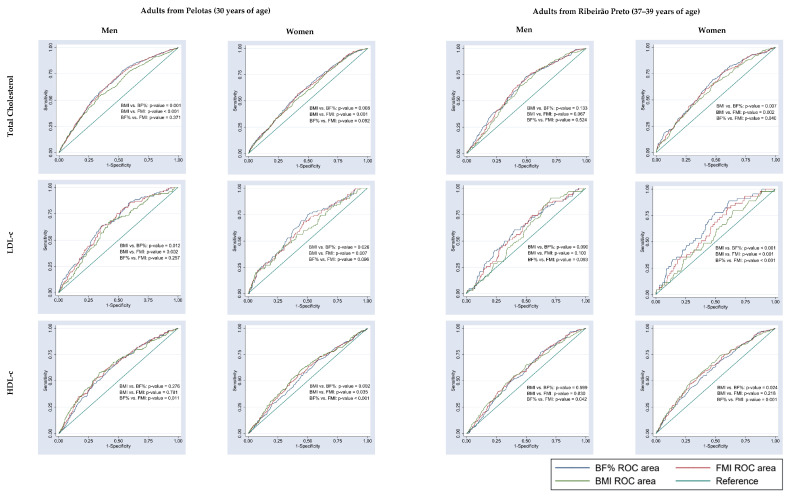
Comparison of the AUC values of body fat percentage (BF%), fat mass index (FMI) and body mass index (BMI) to predict cardiometabolic risk factors (total cholesterol, LDL-c and HDL-c) in adults from Pelotas (RS), 2012/2013, and from Ribeirão Preto (SP), 2016/2017.

Comparison of the AUC values of BMI versus BF% and BMI versus FMI revealed no significant difference for blood glucose, triglycerides or C-reactive protein in either men or women from the two cities (Figure 3 and Figure 4). In adults from Pelotas, the AUC values of BMI did not differ from those of BF% or FMI in identifying altered glycated hemoglobin in either sex, altered blood pressure in women or reduced HDL-c in men (Figure 3, Figure 4 and Figure 5). Similarly, in adults from Ribeirão Preto, the AUC values of BMI did not differ from those of BF% or FMI in identifying altered blood pressure and having three or more cardiometabolic risk factors in either sex (Figure 3 and Figure 4). Only men showed no differences in the AUC values of BMI versus BF% and BMI versus FMI for total cholesterol, LDL-c, HDL-c and glycated hemoglobin (Figure 4 and Figure 5).

## 4. Discussion

The cut-off points for BF%, FMI and BMI varied across the cardiometabolic risk factors and differed between sexes and ages. In general, the AUC values of these indicators were similar and demonstrated low-to-acceptable discriminatory power in predicting the cardiometabolic risk factors. Furthermore, the results indicate that the AUC of BMI did not significantly differ from the AUC of BF% and FMI in detecting most of the cardiometabolic risk factors. When compared to the cut-off established by the WHO [2] for identifying obesity, the BMI cut-off values obtained in this study were lower for both sexes in the two cities.

Considering the influence of age on body composition, the analyses in this study were stratified by cohort, as the cohorts consisted of different age groups. It is well established in the literature that body weight and fat mass increase with age. The estimated rate of fat mass gain in adulthood is 0.37 kg/year in men and 0.41 kg/year in women [24]. Moreover, a study of Norwegian adults showed significant increases in BMI for both sexes over an 11-year period [25].

The BF% cut-off points of our study are similar to those reported by Ramírez-Vélez et al. [26] for Colombian university students with a mean age of 20.6 years, by Pasdar et al. [7] for Iranian adults ranging in age from 35 to 65 years and by Macek et al. [10] for a sample of Polish adults with a mean age of 55.1 years. However, a study involving Mexican adults aged 20 to 65 years reported higher BF% cut-off points [27]. On the other hand, studies on Chinese adults aged 20 to 79 years [13] and 20 to 80 years [28] and on Polish adults aged 37 to 66 years [29] and with a mean age of 55.7 years [30] identified lower cut-off points than those obtained in our study.

Similar to BF%, no specific cut-off points for FMI have been defined for obesity diagnosis. Ramírez-Vélez et al. [26] and Pasdar et al. [7] reported results similar to those obtained here. A study of Chinese adults [10] showed slight differences in FMI cut-off values between sexes and were similar to our results only for men.

Regarding BMI, the cut-off point of ≥30.0 kg/m^2^ to classify obesity in Asian populations (defined by the WHO for individuals ≥ 18 years) has been revised and reduced. However, BMI cut-off points for defining obesity in other populations have not yet been revised [31]. Studies have evaluated BMI cut-off points to screen for cardiometabolic risk factors in adults from different countries [10,29,30,32,33,34,35,36,37,38,39,40,41] and consistently reported lower cut-off points for obesity compared to the WHO [2] for both sexes. Nevertheless, one study of Jordanian adults with a mean age of 43.8 years identified the same BMI cut-off for predicting diabetes and hypertension in women as recommended by the WHO [2] but a lower cut-off (27.0 kg/m^2^) for men [42].

These suggested reductions in BMI cut-off points may be attributed to the differences between the current context and the scenario in which the traditional cut-off points were established. Assessments based on BMI are supported by the assumption that an increase in BMI is accompanied by an increase in BF%. In fact, there is a high probability that an individual with a high BMI has high BF%. However, as emphasized by Carpenter et al. [43], the increase in sedentary behavior has made individuals susceptible to the accumulation of body fat without necessarily exhibiting significant changes in body weight. Thus, the impact of a sedentary lifestyle on the relationship between fat mass and lean mass, with an increase in fat mass at the expense of a reduction in the latter, may have contributed to the decline in the diagnostic capacity of BMI over time. Considering the above and the available evidence, it may be necessary to revise BMI cut-off points by taking into account the current context, especially regarding the proportion of body compartments (fat mass and lean mass). However, it is important to interpret our results with caution due to the cross-sectional design of the study. Longitudinal studies may, therefore, be useful to observe these differences in body proportions over time. Within this context, it should be noted the BMI cut-off points proposed by the WHO [2] were based on longitudinal studies with long follow-up periods for predicting mortality risk.

This comparison of studies reveals the existence of a certain heterogeneity among the findings. This could be attributed to ethnic, racial and age differences between the populations under study [44], as well as variations in the methods used to assess body composition and establish cut-off points, differences in the diagnostic criteria used to classify cardiometabolic risk factors and disparities in prevalence among the evaluated samples. However, despite the observed differences in cut-off points for predicting cardiometabolic risk factors across studies, a certain consistency in the results can be observed. The cut-off points for BF% and FMI were lower in men than women and, in most studies, the BMI cut-off values were lower than those recommended by the WHO [2]. In fact, women generally have higher BF% than men, irrespective of age and ethnic group, and women with the same BMI as men tend to have a higher amount of body fat [45]. The differences in both the content and the distribution of body fat between sexes are well documented in the literature and are attributed to various physiological mechanisms involving endocrine and metabolic aspects [46].

The three indicators (BF%, FMI and BMI) showed low-to-acceptable discriminatory power in identifying individuals with cardiometabolic alterations [23]. Additionally, the sensitivity of the three indicators showed minimal differences and was generally lower than 70%. In terms of comparing the AUC values of the three indicators, we expected that FMI would demonstrate superior discriminatory power compared to the other indicators for most of the risk factors. This expectation was based on the fact that FMI, being a direct measure of body fat, also takes into account the differences in BF% associated with height. This observation was supported by Liu et al. [13], who found that FMI had a greater AUC than BMI and BF% in both sexes when predicting metabolic syndrome.

An important finding of the present study is that BMI exhibited comparable ability to identify individuals with altered cardiometabolic risk factors as compared to BF% or FMI, which are indicators directly considering body fat. This finding aligns with other studies. Bosy-Westphal et al. [47] conducted a study involving adults aged 18 to 84 years, utilizing air displacement plethysmography for BF% measurement and demonstrated that, at the population level, BMI is a suitable index for assessing obesity-related metabolic risk and is not inferior to direct body fat measurement. Gutiérrez-Rojas et al. [48], in a study of Mexican adults with a mean age of 43.3 years, found no significant differences between BMI and FMI in predicting the degree of obesity and metabolic alterations. Furthermore, Ortega et al. [8] observed that BMI was a stronger predictor of cardiovascular mortality compared to measures of total body fat (BF% and FMI). These authors also suggested that BMI may be clinically important, or even more important than body fat indices assessed by more accurate and expensive methods.

The similarity found among the AUC values of BMI, BF% and FMI indicates that the limitation is not solely related to BMI itself, but to the currently recommended cut-off point, which should be lower. In a recent study, De Oliveira et al. [49] demonstrated that reducing the BMI cut-off points used to identify obesity in adolescents and young adults enhanced their sensitivity without compromising specificity.

The results of the present study have important implications for decision-making in clinical practice. Considering that there was no difference in the diagnostic performance of the three evaluated indicators and none of them showed excellent performance, in the absence of more sophisticated methods, the BMI cut-off points identified in our study can be useful in clinical settings for screening cardiometabolic risk factors in Brazilian adults. At a collective level, it is suggested that the currently used WHO cut-off points in Brazil be revised, and lower values, as identified in our study, be used to guide public health interventions.

Our study has some limitations. Due to loss of participants before follow-up, the evaluated samples may not be representative of the original cohorts regarding certain participant characteristics, and the possibility of selection bias cannot be ruled out. However, such losses are common in cohort studies with long-term follow-ups, as in the cohorts assessed in our study. Nevertheless, it was still possible to evaluate a sufficiently large sample to ensure high statistical power in the analyses conducted. The potential for measurement bias in obtaining anthropometric and body composition measures is unlikely, as these were obtained using accurate and calibrated equipment and performed by trained professionals. On the other hand, regarding the biochemical measurements*,* one limitation of the present study is that the blood collection for the biochemical analyses was not preceded by fasting, as non-fasting blood glucose cut-offs are not available. However, it has been suggested that, in normoglycemic individuals, blood glucose levels usually do not exceed 100 mg/dL regardless of fasting status [50]. In this sense, it is suggested to conduct research with representative samples in which blood collection for biochemical analysis of the participants is preceded by fasting, ensuring the acquisition of more accurate biochemical data and greater external validity of the results.

The strengths of the study include the assessment of body fat by air displacement plethysmography (Bod Pod^®^). Studies have shown that air displacement plethysmography is a rapid, safe and valid technique for assessing body composition, providing relatively accurate results [51,52]. Another strength is the evaluation of large samples, including participants of different age groups, such as young and middle-aged adults. Additionally, to our knowledge, this study is the first to evaluate cut-off values of BF%, FMI and BMI for predicting cardiometabolic risk factors in adults from two Brazilian cities.

## 5. Conclusions

In conclusion, our results showed that the identified cut-off points had low but acceptable discriminatory power. The BMI cut-off points were lower than those recommended by the WHO [2] for obesity. As a simple and low-cost measure, BMI exhibited diagnostic capacity comparable to that of direct body fat indicators with lower cut-offs, indicating its usefulness as a screening tool for cardiometabolic disorders.

## Figures and Tables

**Table 1:** Descriptive characteristics of adults from Pelotas (RS) in 2012/2013 and from Ribeirão Preto (SP) in 2016/2017.

Variables	Adults from Pelotas (30 Years of Age)					Adults from Ribeirão Preto (37–39 Years of Age)				
	n	Men	n	Women	*p*-Value *	n	Men	n	Women	*p*-Value *
Weight (kg)	1735	80.2 (70.6–90.7) ^a^	1782	65.9 (58.0–78.0) ^a^	<0.001	808	88.9 (87.8–90.0) ^b^	888	75.4 (74.3–76.5) ^b^	<0.001
Height (cm)	1735	174.4 (174.1–174.7) ^b^	1782	161.4(161.1–161.7) ^b^	<0.001	808	175.4 (174.9–175.9) ^b^	888	162.6 (162.1–163.0) ^b^	<0.001
BMI (kg/m^2^)	1735	26.3 (23.7–29.5) ^a^	1782	25.3 (22.5–29.7) ^a^	<0.001	808	28.3 (25.6–31.6) ^a^	888	27.4 (24.0–32.0) ^a^	0.001
Non-obese, n (%)		1351 (77.9)		1359 (76.3)	0.258		522 (64.6)		587 (66.1)	0.517
Obese, n (%)		384 (22.1)		423 (23.7)			286 (35.4)		301 (33.9)	
FMI (kg/m^2^)	1735	6.5 (4.3–8.9) ^a^	1782	9.5 (7.0–12.8) ^a^	<0.001	808	7.5 (5.2–10.0) ^a^	888	10.5 (8.0–14.1) ^a^	<0.001
BF%	1735	24.6 (24.2–25.0) ^b^	1782	37.4 (37.0–37.7) ^b^	<0.001	808	25.9 (25.4–26.5) ^b^	888	38.3 (37.7–38.9) ^b^	<0.001
Systolic blood pressure (mmHg)	1735	127.0 (119.5–135.5) ^a^	1780	113.5 (106.5–121)	<0.001	808	127.7(119.5–136) ^a^	887	115 (107.5–124.5) ^a^	<0.001
Normal, n (%)		1001 (57.7)		1585 (89.0)	<0.001		452 (55.9)		704 (79.4)	<0.001
Altered, n (%)		734 (42.3)		195 (11.0)			356 (44.1)		183 (20.6)	
Diastolic blood pressure (mmHg)	1735	76.0 (70.5–82.5) ^a^	1780	73.0 (68.0–79.5) ^a^	<0.001	808	79.5 (73.5–86.5) ^a^	887	75.0 (68.5–82.0) ^a^	<0.001
Normal, n (%)		1387 (79.9)		1547 (86.9)	<0.001		527 (65.2)		685 (77.2)	<0.001
Altered, n (%)		348 (20.1)		233 (13.1)			281 (34.8)		202 (22.8)	
Blood pressure (mmHg)	1735		1780			808		887		
Normal, n (%)		963 (55.5)		1.504 (84.5)	<0.001		397 (49.1)		653 (73.6)	<0.001
Altered, n (%)		772 (44.5)		276 (15.5)			411 (50.9)		234 (26.4)	
Blood glucose (mg/dL)	1717	88.0 (81.0–97.0) ^a^	1771	84.0 (77.0–92.0) ^a^	<0.001	805	91.0 (82.0–103.0) ^a^	887	86.0 (78.0–97.0) ^a^	<0.001
Normal, n (%)		1346 (78.4)		1549 (87.5)	<0.001		552 (68.6)		679 (76.6)	<0.001
Altered, n (%)		371 (21.6)		222 (12.5)			253 (31.4)		208 (23.4)	
Triglycerides (mg/dL)	1717	106.0 (73.0–168) ^a^	1771	86.0 (64.0–123.0) ^a^	<0.001	803	163.0(105.0–247.0) ^a^	886	101.0 (72.0–148.0) ^a^	<0.001
Normal, n (%)		1307 (76.1)		1596 (90.1)	<0.001		429 (53.4)		724 (81.7)	<0.001
Altered, n (%)		410 (23.9)		175 (9.9)			374 (46.6)		162 (18.3)	
Total cholesterol (mg/dL)	1717	189.0 (166.0–217) ^a^	1771	186.0 (165.0–213) ^a^	0.017	803	184.0 (160.0–210.0) ^a^	886	174.0 (153.0–196.0) ^a^	<0.001
Normal, n (%)		855 (49.8)		963 (54.4)	0.007		439 (54.7)		609 (68.2)	<0.001
Altered, n (%)		862 (50.2)		808 (45.6)			364 (45.3)		282 (31.8)	
LDL-c (mg/dL)	1717	112.3 (110.9–113.7) ^b^	1771	106.6 (105.3–107.9) ^b^	<0.001	748	105.0 (85.0–127.0) ^a^	874	99.0 (83.0–120.0) ^a^	0.002
Normal, n (%)		1596 (92.9)		1686 (95.2)	0.005		682 (91.2)		829 (94.9)	0.003
Altered, n (%)		121 (7.1)		85 (4.8)			66 (8.8)		45 (5.1)	
HDL-c (mg/dL)	1717	53.8 (53.2–54.4) ^b^	1771	63.4 (62.8–64.1) ^b^	<0.001	802	41.6 (35.3–47.3) ^a^	886	48.0 (40.6–57.5) ^a^	<0.001
Normal, n (%)		1528 (89.0)		1492 (84.2)	<0.001		441 (55.0)		382 (43.1)	<0.001
Reduced, n (%)		189 (11.0)		279 (15.8)			361 (45.0)		504 (56.9)	
C-reactive protein (mg/dL)	1717	0.1 (0.1–0.3) ^a^	1771	0.3 (0.1–0.7) ^a^	<0.001	801	0.1 (0.1–0.3) ^a^	885	0.3 (0.1–0.6) ^a^	<0.001
Normal, n (%)		1686 (98.2)		1703 (96.2)	<0.001		790 (98.6)		850 (96.1)	0.001
Altered, n (%)		31 (1.8)		68 (3.8)			11 (1.4)		35 (3.9)	
Glycated hemoglobin (%)	1718	5.1 (4.9–5.3) ^a^	1772	5.1 (4.9–5.3) ^a^	0.012	805	5.3 (5.0–5.6) ^a^	883	5.2 (5.0–5.5) ^a^	0.002
Normal, n (%)		1570 (91.4)		1614 (91.1)	0.753		676 (84.0)		775 (87.8)	0.025
Altered, n (%)		148 (8.6)		158 (8.9)			129 (16.0)		108 (12.2)	
Three or more cardiometabolic risk factors, n (%)	1735	452 (26.1)	1782	216 (12.1)	<0.001	808	386 (47.8)	888	243 (27.4)	<0.001

BMI: body mass index; FMI: fat mass index; BF%: body fat percentage; ^a^ Median and interquartile range; ^b^ Mean and 95% confidence interval. * *p*-Value for differences between sexes (continuous variables: Student’s *t*-test for variables with a normal distribution and Mann–Whitney test for variables with a non-normal distribution. Categorical variables: chi-squared test).

**Table 2:** Definition of cut-off points for body fat percentage, fat mass index and body mass index in adults from Pelotas (RS), 2012/2013, and from Ribeirão Preto (SP), 2016/2017.

Cardiometabolic Risk Factors	Adults from Pelotas (30 Years of Age)						Adults from Ribeirão Preto (37–39 Years of Age)					
	Men			Women			Men			Women		
	BF%	FMI (kg/m^2^)	BMI (kg/m^2^)	BF%	FMI (kg/m^2^)	BMI (kg/m^2^)	BF%	FMI (kg/m^2^)	BMI (kg/m^2^)	BF%	FMI (kg/m^2^)	BMI (kg/m^2^)
**Blood pressure ^1^**—*Pelotas (1735 men and 1780 women) and Ribeirão Preto (808 men and 887 women)*												
Cut-off	25.5	6.7	26.4	39.1	10.4	26.4	26.1	7.5	28.3	40.5	11.6	28.6
Sensitivity (%)	61.1	62.8	64.2	61.6	62.0	61.6	61.6	62.8	64.5	67.5	67.9	68.4
Specificity (%)	61.1	61.5	63.3	60.2	62.0	61.5	60.4	62.2	62.5	67.2	67.8	66.3
AUC (95%CI)	0.652 (0.626;0.678)	0.668 (0.642;0.694)	0.680 (0.654;0.705)	0.647 (0.609;0.686)	0.652 (0.614;0.691)	0.653 (0.614;0.692)	0.651 (0.614;0.689)	0.666 (0.629;0.703)	0.672 (0.636;0.709)	0.729 (0.690;0.767)	0.745 (0.707;0.783)	0.746 (0.708;0.784)
Discriminant Power (AUC)	Acceptable						Acceptable					
**Blood glucose ^2^**—*Pelotas (1717 men and 1771 women) and Ribeirão Preto (805 men and 887 women)*												
Cut-off	26.3	7.0	26.8	38.4	10.0	25.9	26.6	7.6	28.6	39.7	11.2	28.1
Sensitivity (%)	59.3	59.3	59.3	56.8	56.8	56.3	56.5	56.9	57.7	60.1	60.1	58.6
Specificity (%)	58.2	59.0	58.8	55.7	56.6	56.2	55.8	55.1	56.2	59.8	59.9	57.9
AUC (95%CI)	0.609 (0.577;0.642)	0.617 (0.585;0.650)	0.618 (0.585;0.650)	0.597 (0.557;0.637)	0.596 (0.555;0.636)	0.587 (0.546;0.629)	0.592 (0.550;0.635)	0.593 (0.550;0.636)	0.581 (0.537;0.624)	0.636 (0.594;0.678)	0.640 (0.598;0.683)	0.633 (0.589;0.676)
Discriminant Power (AUC)	Low						Low					
**Triglycerides ^3^**—*Pelotas (1717 men and 1771 women) and Ribeirão Preto (803 men and 886 women)*												
Cut-off	26.9	7.2	27.1	39.6	10.6	26.9	26.3	7.3	28.4	40.0	11.5	28.5
Sensitivity (%)	64.9	65.8	65.4	62.3	62.3	63.4	61.0	63.6	61.2	61.1	61.7	62.3
Specificity (%)	64.4	64.5	64.3	61.6	62.2	63.0	60.8	62.0	60.4	59.9	61.6	60.6
AUC (95%CI)	0.697 (0.670;0.724)	0.708 (0.681;0.735)	0.702 (0.674;0.729)	0.684 (0.645;0.724)	0.693 (0.652;0.733)	0.684 (0.641;0.727)	0.650 (0.612;0.688)	0.661 (0.624;0.698)	0.654 (0.616;0.691)	0.655 (0.611;0.698)	0.662 (0.619;0.706)	0.657 (0.612;0.702)
Discriminant Power (AUC)	Acceptable						Acceptable					
**Total cholesterol ^4^**—*Pelotas (1717 men and 1771 women) and Ribeirão Preto (803 men and 886 women)*												
Cut-off	25.2	6.5	26.3	37.4	9.5	25.4	26.3	7.6	28.4	39.3	11.0	27.8
Sensitivity (%)	61.2	61.6	59.3	57.4	57.5	55.2	59.6	61.0	58.8	59.6	59.6	57.1
Specificity (%)	61.2	61.2	58.2	56.5	57.4	55.1	59.2	60.8	57.9	59.6	58.9	56.8
AUC (95%CI)	0.655 (0.629;0.680)	0.652 (0.626;0.678)	0.626 (0.599;0.652)	0.600 (0.573;0.626)	0.595 (0.568;0.621)	0.583 (0.556;0.609)	0.631 (0.593;0.669)	0.628 (0.590;0.667)	0.612 (0.573;0.651)	0.635 (0.597;0.674)	0.625 (0.586;0.663)	0.607 (0.567;0.646)
Discriminant Power (AUC)	Acceptable		Low				Low					
**LDL-c ^5^**—*Pelotas (1717 men and 1771 women) and Ribeirão Preto (748 men and 874 women)*												
Cut-off	27.8	7.5	27.3	39.7	10.3	26.1	27.8	7.7	28.5	40.6	11.1	27.5
Sensitivity (%)	63.6	63.6	61.2	61.2	58.8	56.5	60.6	56.1	53.0	60.0	55.6	55.6
Specificity (%)	63.3	63.1	61.1	61.1	58.2	56.3	60.0	55.7	52.9	59.9	55.0	51.0
AUC (95%CI)	0.666 (0.619;0.713)	0.660 (0.613;0.706)	0.625 (0.576;0.673)	0.638 (0.580;0.697)	0.625 (0.565;0.684)	0.601 (0.538;0.663)	0.614 (0.544;0.683)	0.601 (0.535;0.668)	0.573 (0.510;0.637)	0.664 (0.589;0.740)	0.618 (0.541;0.695)	0.565 (0.483;0.647)
Discriminant Power (AUC)	Acceptable		Low				Low			Acceptable	Low	
**HDL-c ^6^**—*Pelotas (1717 men and 1771 women) and Ribeirão Preto (802 men and 886 women)*												
Cut-off	26.8	7.2	27.1	38.1	9.9	26.0	26.2	7.5	28.4	38.5	10.2	27.2
Sensitivity (%)	59.3	60.8	60.3	54.8	56.3	57.7	55.1	56.8	56.5	57.7	59.3	59.9
Specificity (%)	58.8	60.1	59.6	54.5	56.0	57.5	54.4	55.1	56.0	57.1	59.2	59.2
AUC (95%CI)	0.618 (0.577;0.659)	0.630 (0.588;0.672)	0.633 (0.589;0.676)	0.558 (0.522;0.594)	0.574 (0.538;0.611)	0.585 (0.548;0.621)	0.580 (0.540;0.619)	0.588 (0.549;0.628)	0.587 (0.547;0.626)	0.604 (0.567;0.641)	0.620 (0.582;0.657)	0.626 (0.589;0.663)
Discriminant Power (AUC)	Low						Low					
**C-reactive protein ^7^**—*Pelotas (1717 men and 1771 women) and Ribeirão Preto (801 men and 885 women)*												
Cut-off	25.3	6.3	26.7	39.6	10.8	27.2	27.2	7.3	28.3	42.2	12.2	29.6
Sensitivity (%)	51.6	48.4	54.8	63.2	63.2	63.2	54.5	54.5	54.5	68.6	65.7	65.7
Specificity (%)	50.4	47.7	54.0	60.1	63.0	63.2	54.4	48.0	49.1	66.1	65.1	64.7
AUC (95%CI)	0.526 (0.414;0.637)	0.523 (0.409;0.637)	0.523 (0.403;0.644)	0.675 (0.603;0.746)	0.679 (0.607;0.752)	0.673 (0.599;0.748)	0.577 (0.363;0.791)	0.565 (0.344;0.787)	0.539 (0.300;0.779)	0.740 (0.652;0.828)	0.740 (0.657;0.824)	0.738 (0.662;0.815)
Discriminant Power (AUC)	Low			Acceptable			Low			Acceptable		
**Glycated hemoglobin ^8^**—*Pelotas (1718 men and 1772 women) and Ribeirão Preto (805 men and 883 women)*												
Cut-off	25.7	6.7	26.4	37.7	9.8	25.9	27.5	7.8	29.0	40.5	11.8	29.2
Sensitivity (%)	53.4	52.0	51.3	51.9	54.4	55.7	58.9	58.1	58.1	61.1	63.9	65.7
Specificity (%)	52.6	52.0	50.5	51.7	54.3	55.6	58.7	58.0	58.1	60.9	63.7	65.3
AUC (95%CI)	0.539 (0.492;0.587)	0.537 (0.489;0.584)	0.527 (0.478;0.576)	0.545 (0.498;0.592)	0.556 (0.509;0.603)	0.565 (0.518;0.612)	0.614 (0.559;0.667)	0.622 (0.568;0.676)	0.624 (0.570;0.677)	0.638 (0.579;0.696)	0.677 (0.621;0.733)	0.709 (0.656;0.761)
Discriminant Power (AUC)	No discrimination			Low			Low				Acceptable	
**Three or more cardiometabolic risk factors**—*Pelotas (1735 men and 1782 women) and Ribeirão Preto (808 men and 888 women)*												
Cut-off	26.8	7.2	27.0	39.6	10.5	26.9	26.3	7.5	28.4	40.0	11.4	28.5
Sensitivity (%)	65.7	66.1	65.9	63.4	63.0	64.3	61.9	64.2	63.2	65.0	65.4	64.6
Specificity (%)	65.0	66.0	64.8	62.3	62.4	63.9	61.8	64.0	62.3	64.2	65.4	64.5
AUC (95%CI)	0.713 (0.687;0.739)	0.721 (0.695;0.747)	0.707 (0.679;0.734)	0.675 (0.636;0.714)	0.681 (0.641;0.720)	0.673 (0.632;0.714)	0.680 (0.643;0.717)	0.691 (0.655;0.727)	0.683 (0.647;0.720)	0.708 (0.670;0.745)	0.720 (0.683;0.757)	0.717 (0.679;0.755)
Discriminant Power (AUC)	Acceptable						Acceptable					

BF%: body fat percentage; FMI: fat mass index; BMI: body mass index; AUC, area under the receiver operating characteristic (ROC) curve; 95%CI: 95% confidence interval. ^1^ Blood pressure ≥ 130 mmHg and/or ≥ 85 mmHg. ^2^ Blood glucose ≥ 100 mg/dL. ^3^ Triglicerides ≥ 175 mg/dL. ^4^ Total cholesterol ≥ 190 mg/dL. LDL-c: low-density lipoprotein cholesterol; HDL-c: high-density lipoprotein cholesterol.; ^5^ LDL-c ≥ 160 mg/dL. ^6^ HDL-c < 40 mg/dL (men) and < 50 mg/dL (women). ^7^ C-reactive protein > 2 mg/dL. ^8^ Glycated hemoglobin ≥ 5.7%.

## Data Availability

The data presented in this study are available on request from the corresponding author.
